# Protocol to collect late latency auditory evoked potentials

**DOI:** 10.1016/S1808-8694(15)30553-X

**Published:** 2015-10-19

**Authors:** Luzia Maria Pozzobom Ventura, Kátia de Freitas Alvarenga, Orozimbo Alves Costa Filho

**Affiliations:** 1 Master's degree in speech therapy, Dentistry School of Bauru, Sao Paulo University - USP. Speech therapist at the Audiology Research Center, Craniofacial Anomaly Rehabilitation Hospital, USP; 2 Postdoctoral degree in electrophysiology, University of Michigan, and in child audiological assessment, University of Manchester. Associate professor at the Speech Therapy Department, Dentistry School of Bauru, USP; 3 Doctoral degree in otology science, Pontifical Catholic University of Sao Paulo. Full professor at the Speech Therapy Department, Dentistry School of Bauru, USP

**Keywords:** protocols, evoked potentials, auditory

## Abstract

Long Latency Auditory Evoked Potentials (LLAEP) represents a number of electrical changes occurring in the central nervous system, resulting from stimulation of the auditory sensorial pathways. Many studies approach the use of these potentials controlling the artifact created by eye movement with the use of equipment with a large number of channels. However, what happens is very different in Brazilian clinical practice, where the equipment used has a very limited number of channels.

**Aim:**

to compare the two methods used to control the artifacts created by eye movements during LLAEP capture using two recording channels.

**Materials and Methods:**

this is a prospective study with the application of two LLAEP capturing methods (eye artifact subtraction and rejection limit control) in 10 normal hearing individuals.

**Results:**

we did not observe statistically significant differences concerning the latency values obtained with the use of both methods, only concerning amplitude values.

**Conclusion:**

both methods were efficient to capture the LLAEP and to control the eye movement artifact. The rejection limit control method produced greater amplitude values.

## INTRODUCTION

Many systems generate long-latency auditory evoked potentials (LLAEPs), in particular the thalamocortical and corticocortical auditory pathways, the primary auditory cortex, and associative cortical areas.^1^ LLAEPs are represented on electroencephalogram tracings as a series of peaks that include the P1, N1 and P2 components.

Two Brazilian studies have applied LLAEPs testing in their methods to characterize the maturation of the auditory system;^2^,^3^ these studies showed that maturation is reflected in age-related amplitude and latency variations of P1, N1 and P2.

Variables to be controlled in LLAEP recordings are sleep and sedation, otherwise the amplitude of potentials may be attenuated and poorly reproducible.^4^ Thus, a positive factor for testing short latency potentials in children, namely natural or induced sleep by sedation, introduces an additional variable, the presence of ocular movements, which may generate artifacts that contaminate the tracings of potentials. Thus, in LLAEP testing, controlling ocular movement artifacts is essential. There are many internationally published papers dealing with this issue;^5^, ^6^, ^7^, ^8^, ^9^, ^10^, ^11^, ^12^, ^13^, ^14^, ^15^, ^16^, ^17^, ^18^, ^19^, ^20^, ^21^, ^22^, ^23^, ^24^, ^25^, ^26^, ^27^ in the Brazilian medical literature, however, there is only one paper on this topic.^3^ The arrangement of electrodes is one of the variables to be taken into account for recording ocular movement; the supra- and infraorbital positions are preferred.^3^,^5^, ^6^, ^7^, ^8^, ^9^, ^10^, ^11^, ^12^, ^13^, ^14^ Additionally, other studies have suggested using jointly the electrodes placed on the external end of the eyes.^11^, ^12^, ^13^, ^14^ Other techniques include automatically controlling the artifact, which consists of automatically excluding auditory potentials recorded during ocular movement,^6^,^13^,^15^, ^16^, ^17^, ^18^ eye fixation as a method for minimizing ocular movement,^8^,^19^ and rejecting those recordings with amplitudes that encompass ocular movement;^5^,^20^, ^21^ no detailed explanation, however, is given as to how these techniques are done. In Brazil, fully using the methods presented in the international literature is not feasible, because many recording channels are used for controlling ocular artifacts, and in most cases the devices available in this country have only two recording channels. Thus, an assessment protocol adapted to locally available resources is needed. Therefore, the purpose of this study was to compare two ocular artifact-controlling methods when recording LLAEPs based on the recording system of potentials from two channels.

## METHOD

The Institutional Review Board approved this study (number 99/2006), and subjects provided consent for their participation and dissemination of results, according to the Law 196/96, by signing the free informed consent form.

The series consisted of 10 adults aged from 20 to 31 years, mean age of 25 years and 10 months (±3.36 years); there were five males and five females, all with no audiological complaints or any history of neurological conditions. Subjects were normal hearing, as demonstrated by pure tone audiometry, done with a Madsen Electronics Midimate 622 device in an acoustic booth, and acoustic immittance testing done with a Grason-Stadler GSI device.

LLAEP testing was done with an Intelligent Hearing Systems Smart EP USB Jr two-channel device calibrated in hearing level (dBHL) before the test. Response stimulus and recording parameters were:

– Click stimulus, condensation polarity, 100 μs duration, 526 ms inter-stimulus interval, presented using insertion phones in the right ear, 70 dBHL intensity, and 1.9 stimulus-per-second presentation rate.
– Band pass filter 1 to 30 Hz, 100.0 K gain in both channels, response analysis window –100 ms pre-stimulation to 500 ms post-stimulus. 512 stimuli promediated stimuli were used twice to check double recordings.
– Disposable Meditrace^TM^ 200 ECG electrodes and Tem 20TM conductive gel for EEG, placed after skin cleaning with Nuprep abrasive gel for ECG/EEG. The impedance level was kept from 1-3 Kohms.

With only two recording channels, one was used for recording auditory evoked potentials (Channel A) and the other was used for recording ocular movements and blinking (Channel B). The channel A active electrode was placed on Cz, connected to the preamplifier input (+) and the reference electrode was placed on the right ear lobule (A2), connected to the input (-). The ground electrode was placed on the left ear lobule (A1), connected to the ground position. The channel B active electrode was placed on the left supraorbital position, connected to the preamplifier input (+) and the reference electrode was placed on the left infraorbital position, connected to input (-).

The artifact rejection level was adjusted in channel B to include the ocular movement amplitude and blinking in each subjects, and transposed to channel A to maintain a 30% level of rejected stimuli, to record potentials with a morphology that made it possible to accurately analyze the recording in a feasible time for clinical practice, since lower levels increased the duration of the exam, rendering it impractical.

Having identified the auditory evoke potential, amplitude was established as the difference between the 0.0 μV point (recording baseline) and the maximum positive value, in this case the P1 and P2 components, and the negative value, specifically for the N1 component, measured in μV. P1, N1 and P2 and the latency values were then marked, taking into account the maximum amplitude points.

Testing was done in an acoustically and electrically treated booth; subjects were seated comfortably in a reclining seat, watching a silent movie.

Two ocular movement artifact-cancelling methods were tested, described below:

### Controlling the rejection limit

**Figure 1:**
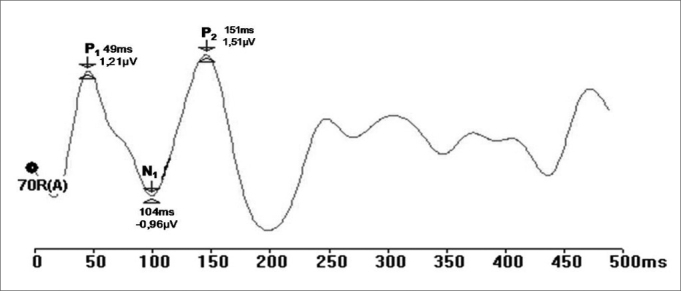
Recording of long latency auditory evoked potentials by the rejection limit control method (ms - milliseconds, mV - microvolt.

Prior recording of ocular movement and blinking was done in channel B to verify its amplitude and set the rejection level for each exam, so that these movements would not be picked up in channel A (and interfere with LLAEP recordings). Thus, auditory evoked potentials were recorded to minimize any interference from ocular movement artifacts (Fig. 1).

### Subtracting the ocular artifact

LLAEPs were recorded in channel A, and ocular movement and blinking were simultaneously recorded in channel B. Next, recording of ocular movements were multiplied by a correction factor (0.1) that had been set in a prior study.^28^ The idea was to minimize any difference between blinking and ocular movement amplitude picked up in channels A and B.

**Figure 2:**
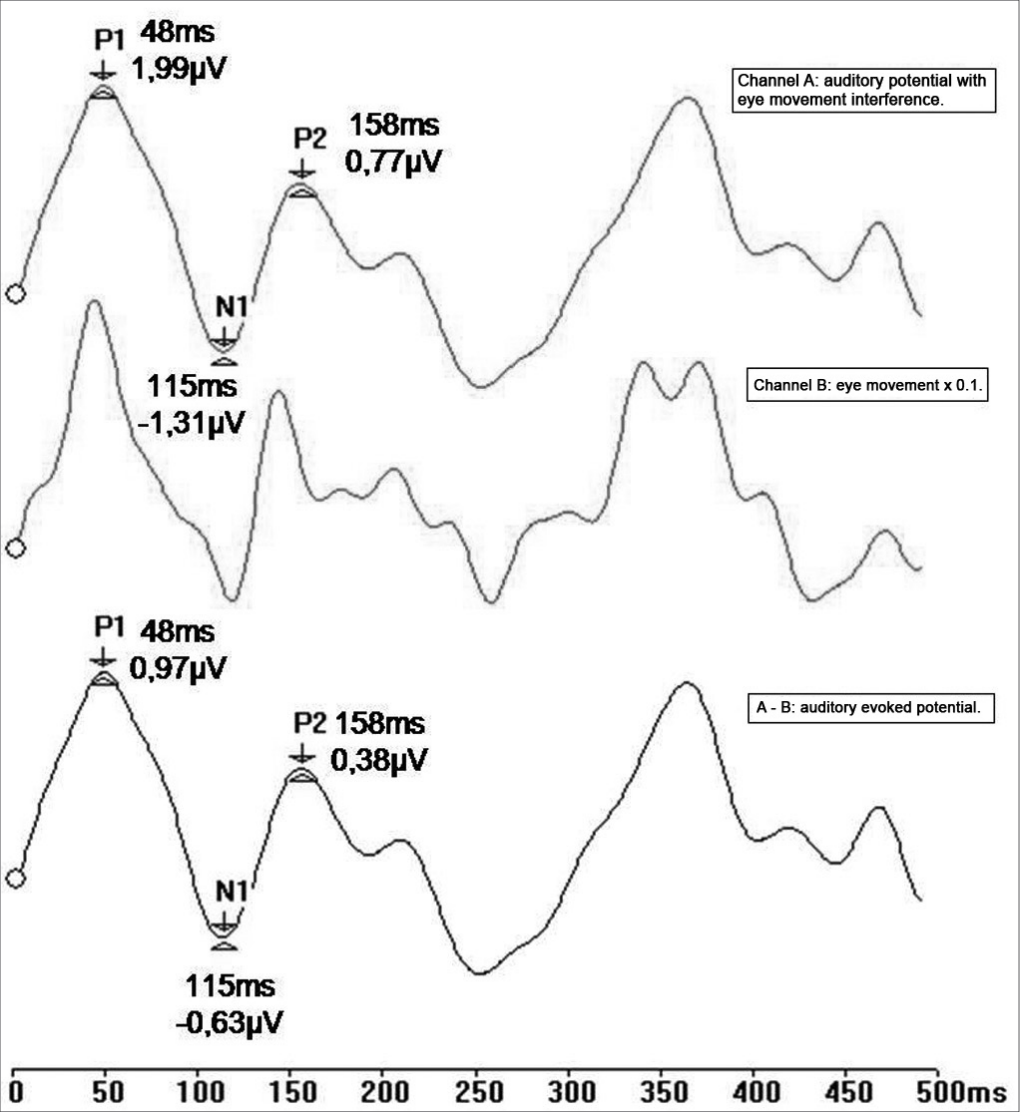
Recording of long latency auditory evoked potentials by the ocular artifact subtraction method (ms - milliseconds, mV - microvolt).

A subtraction process was applied wherein the ocular movement recording was subtracted from the auditory potential recordings (response A-B). An auditory evoked potential recording resulted, which eliminated any interference from ocular movement artifacts; there was also phase correspondence between the potentials (Fig. 2).

LLAEPs were investigated in each study subject using both methods applied twice (1st and 2nd studies) in the same session to verify the test-retest reliability of potentials. The first method varied from subject to subject to control for application order variability.

The statistical study consisted of the paired t test for analyzing latency values and amplitudes among the methods applied in this study. The significance level was p=0.05.

## RESULTS

The results of latency values and LLAEPs amplitudes were analyzed and are shown on tables showing the descriptive statistics (mean, significance level, and p significance level). Table 1 shows the statistical analysis comparing the results from the 1st and 2nd tests of the same method, and the ocular artifact subtraction method and rejection limit control. Table 2 shows the statistical analysis comparing the methods used for controlling the ocular artifact.

**Table 1:** Comparison among latency values, and P1, N1 and P2 amplitude components in each test (1st and 2nd tests) in each method.

Method	Measure	1st Test		2nd Test		p
		Mean	SD	Mean	SD	
Subtraction	LP1	51,22	16,75	49,44	10,19	0,631
	AP_1_	0,36	0,27	0,26	0,17	0,361
	LN_1_	84,25	17,49	81,63	14,07	0,317
	AN_1_	-0,37	0,15	-0,36	0,21	0,903
	LP_2_	140,89	17,69	139,11	19,54	0,775
	AP_2_	0,35	0,12	0,30	0,18	0,407
	LP_1_	50,14	10,9	44,17	10,82	0,433
	AP_1_	0,67	0,32	0,81	0,19	0,169
Rejection	LN_1_	84,56	21,1	84,78	19,36	0,977
	AN_1_	-0,88	0,32	-0,72	0,53	0,393
	LP_2_	139,6	20,57	140,6	23,83	0,906
	AP_2_	0,80	0,39	0,63	0,37	0,298

(L - latency of each component measured in ms, A - amplitude of each component measured in μV, SD - standard deviation, p - significance level).

**Table 2:** Comparison among latency values and P1, N1 and P2 component amplitudes of the different methods.

Measure	Subtraction		Rejection		p
	Mean	SD	Mean	SD	
LP_1_	54,75	13,88	51,63	10,93	0,264
AP_1_	0,34	0,28	0,62	0,34	0,019
LN_1_	84,25	17,49	81,38	20,11	0,482
AN_1_	-0,37	0,15	-0,95	0,27	0,000
LP_2_	140,89	17,69	143,11	18,37	0,350
AP_2_	0,35	0,12	0,81	0,41	0,004

(L - latency of each component measured in ms, A - amplitude of each component measured in μV, SD - standard deviation, p - significance level).

## DISCUSSION

LLAEPs in clinical practice offer a direct non-invasive evaluation of auditory cortical physiology; furthermore, these potentials have been used in several studies investigating the sites where recording are picked up and the maturity level of central auditory structures.

Most of the studies we found were published in the international literature, done with multiple electrodes using multiple channel devices, which we generally do not have available in our context. The degree of alertness affects LLAEPs recordings;4 therefore, several authors recommend controlling ocular movement artifacts in this test. It becomes an ally for reliable recordings by eliminating subjectivity; however, no detailed descriptions were found about this method.^5^, ^6^, ^7^, ^8^, ^9^, ^10^, ^11^, ^12^, ^13^, ^14^, ^15^, ^16^, ^17^, ^18^, ^19^, ^20^, ^21^, ^22^, ^23^, ^24^, ^25^, ^26^, ^27^

Only two studies on the use of these potentials for studying central auditory maturity have been published in Brazilian literature;^2^,^2^ in these cases, two-channel devices were used to demonstrate that, as the auditory system matures, latency values and LLAEP amplitudes change. One of these studies^3^ attempted to control the ocular movement artifact. Thus, a method applicable to local reality was needed for controlling this artifact.

The purpose of this study was not to establish guidelines for latency and amplitude values of LLAEP components in adults, but to describe the recording methods for these procedures and to compare ocular movement control during testing. We intended to apply international parameters used in research adapted to two channel devices, which are generally used in the Brazilian context. The sample size reflected this aim.

Placing channel B electrodes on the supra- and infra-orbital positions was effective for recording ocular movements and blinking, as has been described also by several authors.^5^, ^6^, ^7^, ^8^, ^9^, ^10^, ^11^, ^12^, ^13^, ^14^ Using electrodes placed bilaterally on the external tip of eyes, reported by some authors,^11^, ^12^, ^13^, ^14^ was not done in this study, since there was no electrode for the external ocular region (there were only two channels); this did not affect the quality of ocular movement recordings.

Both methods (rejection limit control and ocular artifact subtraction) enabled us to record LLAEPs, and were easy to apply; both reduced ocular movements.

Table 1 shows the mean amplitude and latency values of each LLAEP components (P1, N1 and P2); no statistically significant differences were seen in the 1st and 2nd tests in both methods (p>0.05), which underlines their test-retest reliability.

Table 2 shows the mean amplitude and latency values of P1, N1 and P2 components, and a comparison between methods (p). There was no statistically significant difference between these methods with regards to latency values, but there was a statistically significant difference (p=0.05) among amplitude values, which were higher when using the rejection limit control method for all components. Higher amplitudes with the rejection limit control method may justify choosing this method in clinical practice, since a higher amplitude often means a better definition of components. The potential can thus be established correctly, resulting in increased accuracy for measuring latency and amplitude values of these potentials.

## CONCLUSION

Both methods effectively recorded LLAEPs and controlled ocular movement artifacts during testing. However, the rejection limit control method yielded the best view of potentials because of higher measured P1, N1 and P2 component amplitude values.
